# Effects of estrogens and bladder inflammation on mitogen-activated protein kinases in lumbosacral dorsal root ganglia from adult female rats

**DOI:** 10.1186/1471-2202-10-156

**Published:** 2009-12-28

**Authors:** Ying Cheng, Janet R Keast

**Affiliations:** 1 Pain Management Research Institute, Kolling Institute of Medical Research, University of Sydney at Royal North Shore Hospital, St Leonards NSW 2065, Australia

## Abstract

**Background:**

Interstitial cystitis is a chronic condition associated with bladder inflammation and, like a number of other chronic pain states, symptoms associated with interstitial cystitis are more common in females and fluctuate during the menstrual cycle. The aim of this study was to determine if estrogens could directly modulate signalling pathways within bladder sensory neurons, such as extracellular signal-related kinase (ERK) and p38 mitogen-activated protein (MAP) kinases. These signalling pathways have been implicated in neuronal plasticity underlying development of inflammatory somatic pain but have not been as extensively investigated in visceral nociceptors. We have focused on lumbosacral dorsal root ganglion (DRG) neurons projecting to pelvic viscera (L1, L2, L6, S1) of adult female Sprague-Dawley rats and performed both *in vitro *and *in vivo *manipulations to compare the effects of short- and long-term changes in estrogen levels on MAPK expression and activation. We have also investigated if prolonged estrogen deprivation influences the effects of lower urinary tract inflammation on MAPK signalling.

**Results:**

In studies of isolated DRG neurons in short-term (overnight) culture, we found that estradiol and estrogen receptor (ER) agonists rapidly stimulated ER-dependent p38 phosphorylation relative to total p38. Examination of DRGs following chronic estrogen deprivation *in vivo *(ovariectomy) showed a parallel increase in total and phosphorylated p38 (relative to β-tubulin). We also observed an increase in ERK1 phosphorylation (relative to total ERK1), but no change in ERK1 expression (relative to β-tubulin). We observed no change in ERK2 expression or phosphorylation. Although ovariectomy increased the level of phosphorylated ERK1 (vs. total ERK1), cyclophosphamide-induced lower urinary tract inflammation did not cause a net increase of either ERK1 or ERK2, or their phosphorylation. Inflammation did, however, cause an increase in p38 protein levels, relative to β-tubulin. Prior ovariectomy did not alter the response to inflammation.

**Conclusions:**

These results provide new insights into the complex effects of estrogens on bladder nociceptor signalling. The diversity of estrogen actions in these ganglia raises the possibility of developing new ways to modulate their function in pelvic hyperactivity or pain states.

## Background

Interstitial cystitis is a chronic condition associated with inflammation of the lower urinary tract, which is more common in women and causes bladder symptoms (e.g., increased urgency and frequency) and pain that are poorly treated [1-3]. While there is considerable debate surrounding the diagnosis and etiology of interstitial cystitis, bladder tissues often show inflammation and ulceration [4,5]. During this period, it is likely that nociceptive C-fibers within the bladder wall become sensitised by neurotrophic factors and other inflammatory mediators [6-9]. Like a number of other chronic pain states, symptoms associated with interstitial cystitis are more common in females and fluctuate during the menstrual cycle [6,10]. Moreover, following ovariectomy, mice develop hyperalgesia and enhanced visceral sensitivity [11,12]. These observations raise the question of the mechanisms by which estrogens could be modulating pain and, more specifically, bladder pain.

Neuroanatomical studies have identified estrogen receptors (ERs) and ER mRNA within many small- and medium-sized lumbosacral dorsal root ganglion (DRG) neurons [13-15]. Evidence supporting a direct effect of estrogens on bladder nociception was provided by Bennett and colleagues, showed that in adult female rat lumbosacral DRG, ERα and ERβ are synthesised by more than half of the bladder-projecting neurons identified by retrograde tracer [16]. Moreover, about one-third of these neurons express both ERs and the nociceptive transducer, transient receptor potential vanilloid receptor 1 (TRPV1), providing a mechanism by which steroid modulation could directly affect bladder pain. More recently, an ERβ-dependent effect of estradiol on nociceptor activity has been identified in adult female rat lumbosacral DRG neurons, where overnight exposure to estradiol or ERβ-agonist powerfully reduces the effects of capsaicin [17]. There is also a large body of evidence supporting rapid actions of estrogens within the nervous system (see review [18]), including the regulation of nociception and pelvic visceral pain. For example, in adult rat lumbosacral DRG neurons, estradiol rapidly induces activation of extracellular signal-regulated kinases (ERK), in turn leading to phosphorylation of cAMP response element binding protein (CREB) [19]. CREB has been strongly linked to neuronal plasticity including long term potentiation [20], so could participate in sensitisation, as demonstrated in the dorsal horn [21].

ERK activation has been causally linked to the development of pain [22], being elevated in nociceptor neurons and spinal cord after inflammatory stimuli and peripheral nerve trauma, including a model of acute visceral pain [23-27]. Chronic visceral inflammation causes a prolonged increase in phosphorylated ERK within the bladder tissues [28]. Moreover, elevated levels of nerve growth factor (NGF) within the inflamed bladder [29] and increased expression of neurotrophic factor receptors in bladder afferent neurons of rats with cystitis [30] could provide a mechanism for mediating this effect on ERK signalling. Irrespective of the mechanism, an important role of mitogen-activated protein (MAP) kinases is indicated by studies showing that intravesical or intrathecal administration of MEK inhibitors increases bladder capacity in rats with cystitis [28,31]. A second family of MAP kinases, the p38 MAP kinases, have been implicated in neuronal plasticity underlying development of inflammatory and neuropathic pain. This pathway can be activated by cytokines, leading to hyperexcitability and repetitive firing of nociceptors in DRG. For example, tissue-derived NGF drives a p38-dependent expression of TRPV1 [32] and p38 causes phosphorylation and increased current density of the sodium channel, Nav1.8 [33]. These actions have not yet been investigated in visceral nociceptors.

While estrogen has been proposed to affect neuroinflammation of the bladder by influencing NGF activity [6], it is also possible that estrogens affect bladder pain by directly modulating signalling pathways within bladder sensory neurons. We have focused on lumbosacral DRG neurons projecting to pelvic viscera (L1, L2, L6, S1) of adult female Sprague-Dawley rats and performed both *in vitro *and *in vivo *manipulations to address the following aims: (i) to determine if estradiol acutely modulates p38 signalling *in vitro*; (ii) to investigate whether chronic estrogen deprivation *in vivo *(ovariectomy) affects p38 or ERK activity, (iii) to determine if chronic bladder inflammation affects p38 or ERK activity and (iv) if prior ovariectomy attenuates or enhances any effects initiated by bladder inflammation. We identified distinct effects of acute and chronic estradiol manipulation on p38 MAP kinase in DRGs. Moreover, while inflammation and ovariectomy both caused some effects on MAP kinases, the nature of these effects differed between p38 and ERK1/2 MAP kinases. These results provide new insights into the complex effects of estrogens on bladder nociceptor signalling.

## Methods

A total of 22 female Sprague-Dawley rats (6-13 weeks old) were used for this study. For *in vitro *studies, animals were 6-7 weeks old at the time of tissue removal. The *in vivo *studies were designed such that the manipulations were commenced at a similar age (6-7 weeks old) and tissues removed at 9-13 weeks of age (depending on treatment group; see below). Rats were obtained from Animal Resources Centre (Perth, Australia) and all procedures were approved by the University of Sydney and Royal North Shore Hospital ethics committees, and conducted in accordance with the Australian Code of Practice for the Care and Use of Animals for Scientific Purposes (National Health and Medical Research Council of Australia) and the National Institutes of Health Guide for the Care and Use of Laboratory Animals. All efforts were made to minimize the number of animals used and their suffering. The estrous cycle of the animals was monitored but not controlled for in these experiments. We did not observe any effects of estrus cycle stage for any of the parameters measured so the results were pooled. Much larger numbers of animals would be required to exclude or confirm an effect of estrus cycle on our measurements.

### *In vitro *studies

Rats were heavily anaesthetized with sodium pentobarbitone (80 mg/kg) then decapitated. Dorsal root ganglia (DRG) were cultured and prepared for Western blotting analyses as described previously [17,19]. Briefly, DRG were dissected from spinal levels L1, L2, L6 and S1, the capsule was quickly removed from each ganglion under a dissecting microscope, ganglia pooled and transferred into modified Tyrodes solution containing (mM) NaCl 130, NaHCO_3 _20, KCl 3, CaCl_2 _4, MgCl_2 _1, HEPES 10, glucose 12 with antibiotic-antimycotic solution. DRGs were then treated with collagenase (Type I, Worthington Biochemical Corporation, West Chester, PA) and trypsin (0.25%, Invitrogen, Mt Waverley, Australia) at 37°C for 1 h, washed, triturated, overlayed on bovine serum albumin (15%, Sigma, Castle Hill, Australia) and centrifuged at 900 rpm for 10 min to remove myelin and debris. The pellet was resuspended with Neurobasal A and B27 (Invitrogen) and for Western blotting studies, neurons plated onto disposable plastic Petri dishes, previously coated with polyornithine (Sigma) and laminin (Invitrogen). For immunohistochemistry, neurons were plated onto glass coverslips similarly coated with polyornithine and laminin. The neurons were maintained at 37°C in a humidified atmosphere containing 5% CO_2 _for 18-24 h before use, to minimize any acute effects of dissociation (e.g. axotomy) on cell signaling [34]. Cultures were then treated with appropriate reagents added to the culture medium as indicated for each experiment in the Results. At the end of treatment, the culture was washed once with cold phosphate-buffered saline (PBS; 0.1 M, pH 7.2), then 200 μl ice-cold T-PER protein extraction reagent (Pierce, Rockford, IL) containing a standard protease inhibitor cocktail (Complete Mini; Roche, Castle Hill, NSW, Australia) and phosphatase inhibitor cocktail (PhosSTOP, Roche) was added. Cultured cells were removed with a cell scraper, briefly sonicated and centrifuged at 10,000 g for 5 min; the supernatant was collected and stored at -20°C for Western blotting. All culture experiments contained an internal control (treated only with vehicle), each sample was tested in duplicate and each experiment was replicated three times.

### Immunohistochemistry

Cultures were fixed in 4% phosphate-buffered paraformaldehyde for 30 min, blocked and permeabilised in phosphate buffered saline (PBS) containing 10% horse serum and 0.1% triton X-100 for 1 h, then incubated with primary antibodies for 2 h. Antisera against the following antigens were used: β-tubulin isotype type III (1:200; host species, mouse; Sigma-Aldrich, Castle Hill, Australia), ERα (1:250; host species, rabbit; Affinity Bioreagents, Golden, CO), ERβ (1:400; host species, chicken; gift from JA Gustafsson, Karolinska Institute, Stockholm). Cultures were then incubated with Cy3- or FITC-tagged secondary antibodies (Jackson Immunoresearch Laboratories, West Grove, PA, USA) for 1 h. DAPI (4',6-diamidino-2-phenylindole dihydrochloride;1 μg/ml, Sigma) was used as a nuclear counterstain. Coverslips were mounted onto slides in 0.5 M bicarbonate-buffered glycerol (pH 8.6) and viewed with an Olympus BX-51 fluorescence microscope. Images (8-bit monochrome) were captured using an RT Spot camera (Diagnostic Instruments, Sterling Heights, MI, USA) and digitised using Image Pro Plus software (Media Cybernetics, Silver Spring, MD, USA). For figure production, minor adjustments were made to contrast and brightness of the entire image, to best represent the immunostaining as viewed under the microscope, using Adobe Photoshop (Version CS3).

### *In vivo *studies

Four groups of rats were studied: control (n = 4), ovariectomy (OVX; n = 4), cyclophosphamide-treated (CYP, n = 5) and ovariectomy prior to cyclophosphamide treatment (OVX-CYP, n = 3). For the ovariectomy group, rats were anaesthetised with ketamine (60 mg/kg i.p.) and xylazine (10 mg/kg i.p.) prior to performing a bilateral ovariectomy. Four weeks later rats were deeply anaesthetised with sodium pentobarbitone (80 mg/kg, i.p.) and DRG (L1, L2, L6, S1) removed and pooled for protein extraction as described above. Tissues from age-matched intact controls were also removed at this time. A second experimental group of rats was treated with cyclophosphamide (CYP) to induce inflammation of the lower urinary tract [35,36]. To administer CYP, animals were briefly anaesthetised with isoflurane then injected with CYP in sterile 0.9% sodium chloride (75 mg/kg, i.p), every three days [30]. On day 10, animals were heavily anaesthetised with sodium pentobarbitone as above, and DRG removed and pooled for protein extraction as above. For the OVX-CYP group, rats were ovariectomised and four weeks later CYP treatment was administered as above, then DRG removed for protein studies. DRGs were placed in 200 μl ice-cold T-PER protein extraction reagent (Pierce, Rockford, IL) containing a standard protease inhibitor cocktail (Complete Mini; Roche, Castle Hill, NSW, Australia) and phosphatase inhibitor cocktail (PhosSTOP, Roche), and homogenized on ice for at least 1 min. Homogenized samples were centrifuged at 10,000 g for 5 min to pellet tissue debris. Supernatant was collected and stored at -20°C in aliquots for Western blotting.

### Western blotting

Protein extracted from freshly dissected or cultured DRGs was mixed with protein loading buffer and heated at 99°C for 3 min, and then kept on ice, until loaded and separated on 12% sodium dodecyl sulfate-polyacrylamide gel (SDS-PAGE; ~10 μg per well). Proteins were then transferred onto PVDF membrane for 2 h on ice and the membrane with transferred proteins then blocked with 5% non-fat milk solution in TBS/Tween (TBST) for 1 hr at room temperature. The membrane was then washed and incubated overnight at 4°C with an antibody raised in rabbit against phospho-p38 (Cell Signaling Technology, Danvers MA; 1:1,000 in TBST with 5% BSA) or phospho-ERK1/2 (Cell Signaling Technology, 1:4,000 in TBST with 5% milk). After washing, the membrane was incubated with horseradish peroxidase-conjugated anti-rabbit IgG (1:50,000 in TBST with 5% milk; Jackson ImmunoResearch) for 1 h at room temperature. A single band (38 kDa) for phospho-p38 or double bands (44 and 42 kDa) for phospho-ERK1/2 were visualized using ECL plus (Amersham, now GE Healthcare). The membranes were stripped with stripping buffer (7 μl 2-mercaptoethanol per ml TBST with 2% SDS) for 30 min and rinsed with TBST for 30 min. The membrane was re-blotted with primary antibody against total p38 (Cell Signaling; 1:1,000 in TBST with 5% BSA) or total ERK1/2 (Cell Signaling; 1:4,000 in TBST with 5% milk) overnight at 4°C, and incubated with horseradish peroxidase-conjugated anti-rabbit IgG as above. A single band (38 kDa) for p38 or double bands (44 and 42 kDa) for ERK1/2 were visualized with ECL plus. Band densities were converted to numerical values using Quantity One (Biorad, Hercules, CA), subtracting background values from an area of gel immediately adjacent to the stained band. Exposure times were chosen to avoid pixel saturation.

Data are expressed as the ratio of phosphorylated p38 or ERK against total p38 or ERK for each sample.

For freshly dissected DRG removed from the four *in vivo *treatment groups, there was sufficient protein available in each sample to allow more than one aliquot to be stored from each animal. This minimised the need for animals in the control group. There were insufficient wells available to run all samples from all groups on one gel, so samples were grouped as follows: Run 1 comprised all samples from control and OVX groups; Run 2 comprised all samples from control, CYP and CYPOVX groups. Because long-term treatments (CYP, OVX, CYPOVX) could affect neuronal structure and growth, we also measured β-tubulin in each of these samples; this also allowed for any minor variations in tissue dissection or preparation. Gels were divided into two sections to allow separate processing for P-p38/p38 (~38 kDA) and β-tubulin (~50 kDa), based on their different migration speed on gels. One membrane section was processed for P-p38 and p38 as described above and the other half was probed for β-tubulin (1:10,000, host mouse; Sigma-Aldrich, Castle Hill, Australia) and anti-mouse IgG (1:50,000; Jackson ImmunoResearch). The results from our *in vivo *experiments have been expressed as raw values for phosphorylated and total ERK (or p38) MAP kinase relative to β-tubulin levels in the same sample. We have also calculated the ratio of phosphorylated to total ERK1, ERK2 or p38 MAP kinase.

### Statistics

All values are expressed as mean ± SE. Analyses were performed with Graphpad Prism (Graphpad Software, San Diego CA). Effects of treatments were compared by unpaired 2-tailed t-test or, for comparison of more than two groups, ANOVA followed by Tukey's test. Significance was accepted if *P *< 0.05.

### Drugs and chemicals

Unless otherwise stated, all reagents were purchased from Sigma-Aldrich. Diarylpropionitrile (DPN), propyl pyrazole triol (PPT) and tamoxifen were purchased from Tocris (Ellisville, MO). ICI 182,780 was a gift of AstraZeneca (North Ryde, Australia).

## Results

### 17β-Estradiol rapidly activated p38 MAPK by an estrogen receptor-dependent mechanism in cultured DRG

**Figure 1:**
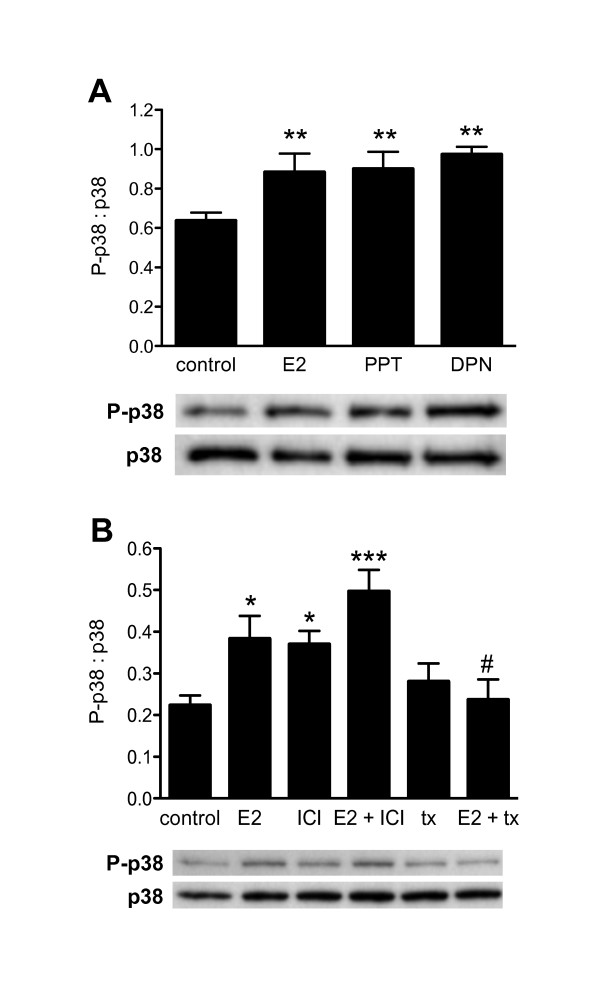
**Phosphorylation of p38 MAP kinase in cultured adult rat DRG neurons following treatment with ER agonists and antagonists (10 min)**. **A**, 17β-Estradiol (E2), PPT and DPN (each 10 nM) all stimulated p38 phosphorylation (separate cultures from 3 rats). A representative Western blot is shown below. **B**, E2 (10 nM) stimulated p38 phosphorylation that was prevented by tamoxifen (tx; 1 μM) and mimicked by ICI182,780 (ICI; 3 μM). The response to E2 with ICI182,780 was greater than with E2 alone. Tamoxifen alone had no effect. Data comprises separate cultures from 3 rats, expressing phosphorylated p38 relative to total p38 protein. A representative Western blot is shown below. * *P *< 0.05, ** *P *< 0.01, *** *P *< 0.001, relative to control; # *P *< 0.05 relative to E2.

Treatment of DRG cultures with 17β-estradiol (10 nM; E2) activated p38 MAPK within 10 minutes (Fig. 1A, B). That is, there was an increase in phospho-p38 compared with total p38 protein. This effect was mimicked by the specific estrogen receptor (ER) agonists, PPT (ERα agonist, 10 nM) and DPN (ERβ agonist, 10 nM) (Fig. 1A). We then tested if the effects of E2 would be inhibited by the pure ER antagonist, ICI182,780 (3 μM) or the estrogen receptor modulator, tamoxifen (1 μM), which antagonises estrogen responses in many tissues. This set of experiments showed that while tamoxifen abolished the response to E2, ICI182,780 not only failed to attenuate the E2 response but itself activated p38 MAPK (Fig. 1B).

**Figure 2:**
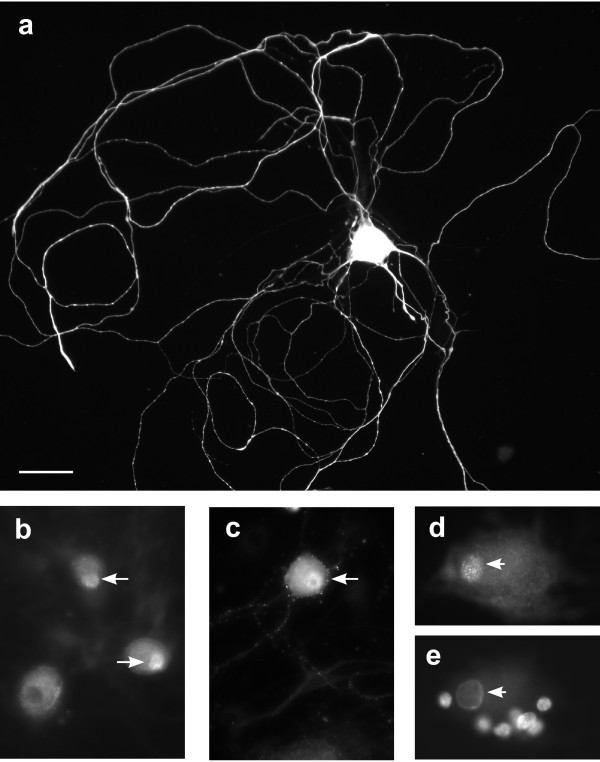
**Adult female rat dorsal root ganglion (DRG) neurons cultured overnight in the absence of serum or added neurotrophic factors**. **a**, Cultured DRG neuron immunostained for β-tubulin, showing extensive neurite growth. **b**, Two neurons showing immunoreactivity for ERα in the nucleus (arrows) and one neuron (bottom left) with no nuclear immunostaining. **c**, Neuron showing bright immunoreactivity for ERβ in the nucleus (arrow), moderate immunoreactivity in the cytoplasm and faint punctate staining in some neurites. **d, e **shows the same neuron, immunostained for ERα **d**) and nuclei labelled with DAPI (**e**); ERα-immunoreactivity is present in the neuronal nucleus (arrow) but not in the closely associated glia. Immunohistochemistry was performed in separate cultures from 3 rats. Calibration bar represents 20 μm (a-c), 10 μm (d, e).

We then examined our cultures immunohistochemically to confirm that estrogen receptors (ERs) were expressed by adult rat DRG neurons in culture and to investigate the possibility of estrogen action on glial cells. Neurons were distinguished from glia by their immunoreactivity for β-tubulin and distinctive properties of their nuclei identified by DAPI. Neuronal nuclei were large with pale DAPI staining, and easily distinguished from glial cell nuclei that were smaller, ovoid and more intensely stained [19]. After 24 h in culture, less than half of the neurons had grown neurites, but many of these possessed long, branching processes (Fig. 2A). ERα-immunoreactivity was identified in many but not all neuronal nuclei (Fig. 2B). Weak ERα-immunoreactivity was also present in the cytoplasm of many somata but was rarely evident within neurites. ERβ-immunoreactivity was identified in many neuronal nuclei and within the soma cytoplasm, and punctate ERβ-immunoreactivity was present in many neurites (Fig. 2C). Neither ERα- nor ERβ-immunoreactivity were evident in glial cells (Fig. 2D, E). We did not quantify the proportion of neurons expressing ERs because many neurons showed relatively dim immunoreactivity and we could not confidently determine how many of these should be considered as genuinely ER-immunoreactive. Together, these two experiments revealed a rapid ER-dependent effect of E2 on p38 activation in DRG neurons and suggest that a novel mechanism underpins this action.

### Chronic estrogen deprivation enhanced p38 MAPK expression and ERK1 phosphorylation

**Figure 3:**
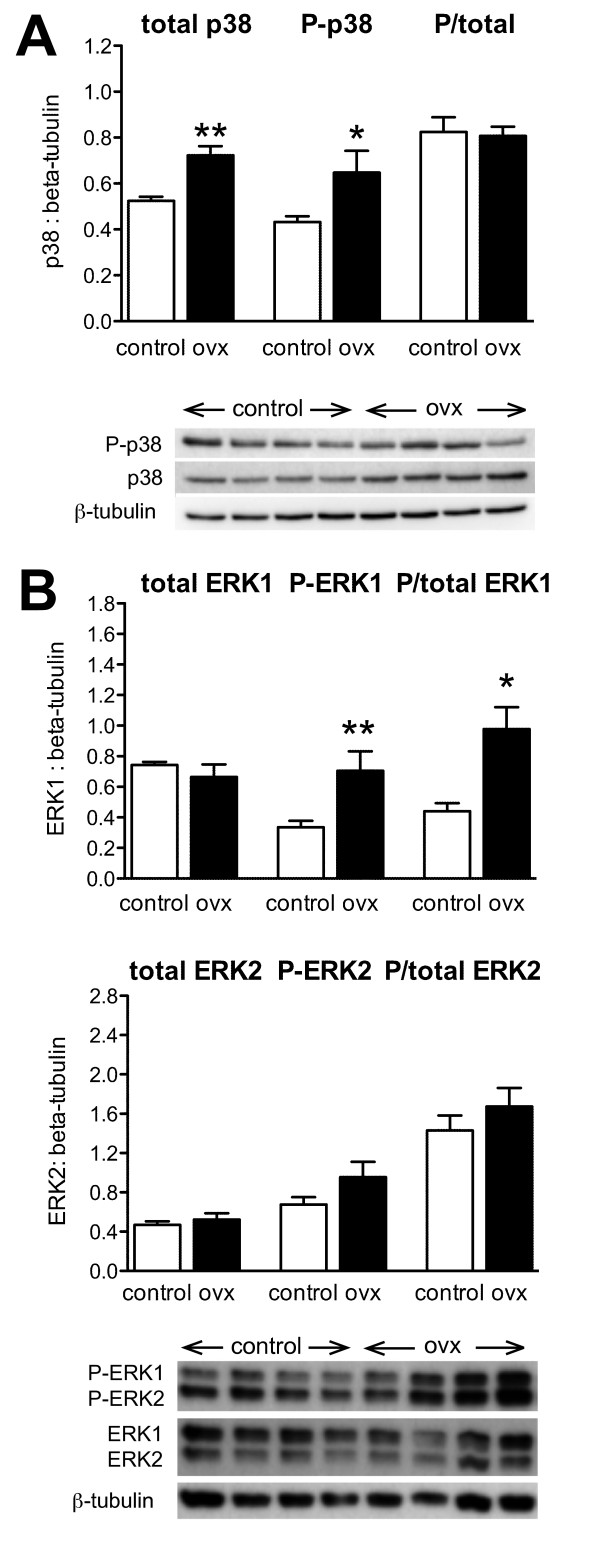
**MAP kinase expression and activation in extracts of DRG removed from control rats and four weeks after ovariectomy (ovx)**. For each type of MAP kinase, the group data is shown with the Western blot for all samples, run at the same time. **A**, Relative to β-tubulin measurements in the same extract, levels of total p38 protein were increased after ovx but there was no evidence for increased phosphorylation. **B**, Relative to β-tubulin measurements in the same culture, there was no effect of ovx on ERK1 expression but phosphorylation was enhanced; in contrast, ovx had no effect on expression or phosphorylation of ERK2. Results represent tissues from 4 rats per group. The same DRG extracts were used for analysis of p38 and ERK1/2. ** *P *< 0.05, ** *P *< 0.01, relative to control.

Although the initial *in vitro *studies revealed rapid-onset activation of p38 MAPK signalling by E2, the long-term effects of changing estrogen exposure *in vivo *are of considerable physiological interest. We therefore compared the effects of prolonged estrogen deprivation (4 weeks post-ovariectomy) on the expression and activation of p38 MAPK within extracts of lumbosacral DRG, focusing on those spinal levels that innervate the urinary bladder (Fig. 3A). Relative to β-tubulin, both total and phosphorylated p38 were increased by ovariectomy, but the ratio of phosphorylated p38 to total p38 protein remained unchanged. In contrast, ovariectomy did enhance ERK1 phosphorylation but had no effect on total ERK1 protein levels (Fig. 3B). Ovariectomy had no significant effect on ERK2 protein levels or ERK2 phosphorylation (Fig. 3B).

### Compared with ovariectomy, lower urinary tract inflammation had similar effects on p38 but not ERK

**Figure 4:**
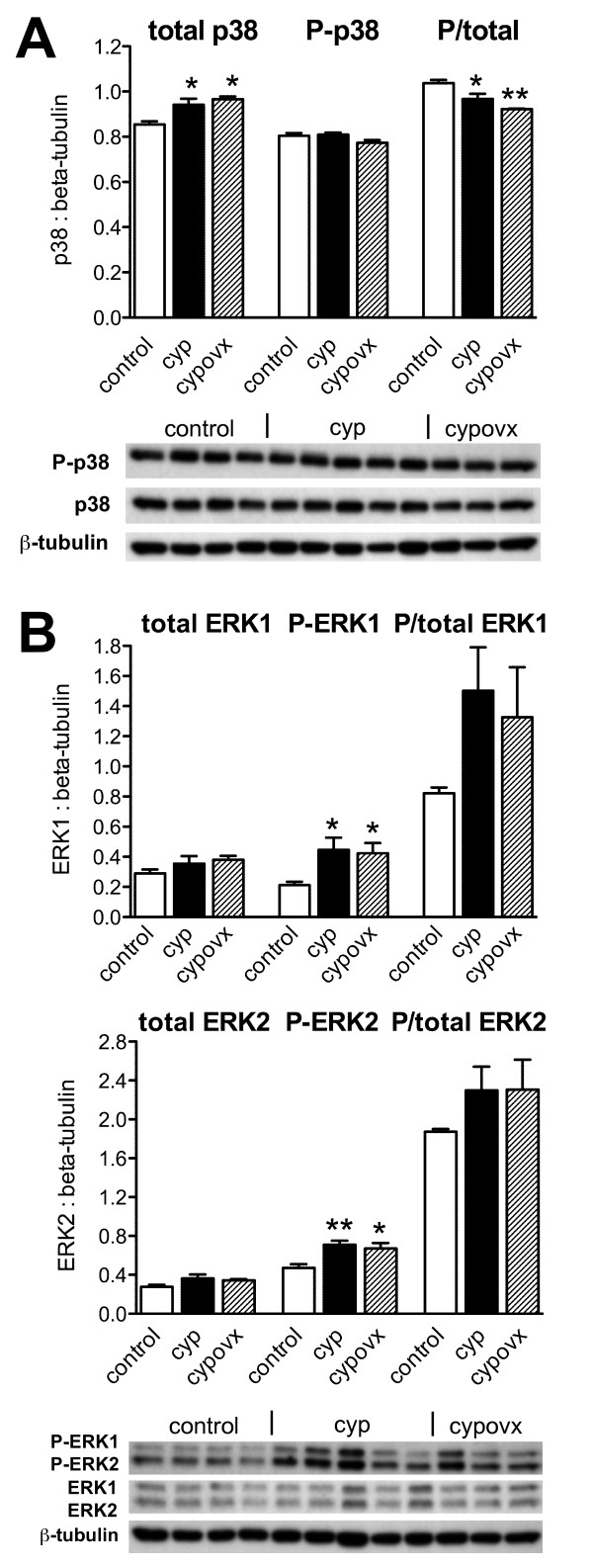
**MAP kinase expression and activation in extracts of DRG removed from control rats, rats with bladder inflammation (cyclophosphamide treatment, cyp) and bladder inflammation evoked four weeks after ovariectomy (cypovx)**. For each MAP kinase, the group data is shown with the Western blot for all samples, run at the same time. **A**, Relative to β-tubulin measurements in the same culture, levels of total p38 protein were increased after cyp and cypovx but there was no evidence for increased phosphorylation. **B**, Relative to β-tubulin measurements in the same culture, there was no effect of cyp or cypovx on ERK1 and ERK2 expression but phosphorylation of each was enhanced. Number of rats per group: control = 4, cyp = 5, cypovx = 3. The same DRG extracts were used for analysis of p38 and ERK1/2. ** *P *< 0.05, ** *P *< 0.01, relative to control.

Chronic lower urinary tract inflammation, i.e. CYP treatment for 10 days, caused a similar effect on p38 MAP kinase as ovariectomy. That is, inflammation alone caused a small increase in p38 protein expression (relative to β-tubulin), however after inflammation there was no parallel increase in p38 phosphorylation (Fig. 4A). Moreover, the inflammation-induced increase in p38 protein was not influenced by prior ovariectomy (Fig. 4A). Inflammation caused an increase in both phospho-ERK1 and phospho-ERK2 but when corrected for loading controls (total ERK1 and ERK2) there was no net effect on phosphorylation of either enzyme (Fig. 4B). These measurements were not significantly affected by prior ovariectomy (Fig. 4A, B).

## Discussion

We have made a number of novel findings that reveal the complexity of estrogenic actions and inflammation in lumbosacral dorsal root ganglia (DRG) and suggest potential strategies for modulating the activity of these neurons in order to attenuate afferent hyperactivity or pain states. In summary, in lumbosacral DRG acute treatment with ER agonists initiated rapid phosphorylation of p38 MAP kinase, whereas prolonged estrogen deprivation *in vivo *(ovariectomy) did not have a long-lasting effect on p38 MAP kinase phosphorylation; instead it caused an increase in p38 expression. It has previously been reported that estradiol causes rapid activation of ERK1/2 in adult rat DRGs [19]. In the present study, prolonged estrogen deprivation *in vivo *had no effect on expression of ERK1/2 in DRGs but caused their prolonged phosphorylation. There were some similarities in the effects of inflammation but the responses were generally smaller or failed to reach statistical significance. It is important to recognise that whereas ovariectomy may influence a broader range of neurons, CYP treatment is likely to target only a small proportion of neurons present in the DRG extracts (i.e., neurons innervating the lower urinary tract), so the actions on MAP kinase signalling less readily identified. The effects of CYP treatment may be larger if bladder-specific neurons could be studied separately, or if upper lumbar and sacral neurons were distinguished.

There are a growing number of examples of rapid actions of estrogen in the nervous system; the mechanisms are diverse and vary considerably between different types of tissues [18,37]. While not yet examined extensively, rapid effects of estrogens have also been reported in DRG, including an action on intracellular calcium levels and ATP-induced calcium currents [38-40], as well as ER-dependent ERK and CREB phosphorylation [19]. In the present study, the effect of estradiol to rapidly stimulate p38 phosphorylation in DRG neurons in short-term culture was mimicked by agonists of ERα and ERβ, which *in vivo *are co-expressed by many lumbosacral DRG neurons [16]. Involvement of ER in this response is also indicated by the blockade by tamoxifen. The transient nature of a rapid response to a single application of estrogen applied *in vitro *may not reflect the nature of estrogen actions *in vivo*, where levels may change more slowly due to changes in circulating hormones (e.g. during the estrous or menstrual cycles) or local production from aromatase-expressing target tissues. Nevertheless these observations are, to our knowledge, the first to indicate the ability of estrogens to active p38 MAP kinase in sensory ganglia.

There are numerous reports of rapid, ER-dependent activation of p38 signalling in non-neuronal cells (e.g. [41-43]). However, in the current study we may have identified a novel mechanism of ER-dependent p38 activation, suggested by the observation that the ER-antagonist, ICI182780, not only mimicked but also enhanced the estrogen response. An "estrogen agonist" effect of ICI182780 has been observed previously in rat DRG, where estradiol inhibition of TRPV1 activity was inhibited by tamoxifen but mimicked by ICI182780 [17]. However, in this earlier study, estrogen agonists and ICI182,780 were each administered to neurons for a much longer period (overnight), which allowed the consideration of a larger variety of possible contributing mechanisms. The "agonist" effect of overnight treatment with ICI182,780 could be due to "tethering" of ER with transcription factors, (e.g., AP-1, STAT5) that affects the nature of subsequent actions with various agents, including ICI182,780 [44]. However, in the current study, our rapid treatment with these agents makes this mechanism less likely so many additional molecular studies will need to be performed to determine the basis of ICI182,780 actions. It is also possible that the effects of ICI182,780 observed here are independent of ER, although this agent is widely considered to be highly specific for these receptors.

Our studies examining the impact of chronic estrogen deprivation and inflammation revealed further complexity in the modulation of MAP kinase signalling pathways in lumbosacral sensory ganglia. Estrogen deprivation for 4 weeks following ovariectomy caused quite different effects on p38, ERK1 and ERK2 signalling pathways from the rapid treatment with estrogen. These showed an upregulation of p38 expression (but not phosphorylation), increased ERK1 phosphorylation (but not expression) and no change in ERK2 expression or phosphorylation. A change in expression of any of the MAP kinases has not commonly been reported after neuronal perturbation and the physiological implications of this are unknown.

The dissimilar actions we observed on each type of MAP kinase are of particular interest in light of a recent study on the effects of bee venom-induced inflammation and hyperalgesia on spinal cord neurons, which showed distinct kinetics of activation for each MAP kinase, i.e. ERK activation occurs rapidly (up to 1 day) after challenge but p38 activation occurs more slowly (1-7 days) [45]. This study also showed a spatial difference in the ERK and p38 activation patterns within the cord. In our study, the greater effects of our manipulations on ERK1 than ERK2 were unexpected, as numerous studies report parallel changes in these two signalling pathways following cell stimulation. However, recent studies have not only identified functional differences in ERK1 and ERK2 [46] and distinct consequences of ERK1 and ERK2 loss [47-49], but also described the structural bases for their functional differences [50]. It is possible that different populations of pelvic nociceptors also show distinct responses.

Previous studies of somatic inflammation have demonstrated an effect on phosphorylation of both ERK and p38 MAP kinases (reviewed in [22]). Our results show that prolonged (10-day) visceral inflammation caused only a very modest effect on phospho-ERK levels in lumbosacral DRG, an effect that did not achieve statistical significance when loading controls (total ERK) were considered. An earlier study using a similar model of bladder inflammation in rats did not detect a comparable change in ERK1/2 phosphorylation in lumbosacral DRG, although they did report a transient (up to 48 h) increase in ERK5 activation [51].

In the current study we could not directly assess the impact of estrogen status on the subsequent response to inflammation because ovariectomy alone caused effects on p38 and ERK MAP kinases. The possibility that these two perturbations activate convergent modulatory mechanisms should be explored further, particularly given the recent observation that some symptoms resembling aspects of interstitial cystitis develop in ERβ-knockout mice [52]. It is also possible that local estrogen production (e.g. in the spinal cord dorsal horn or in peripheral tissues [53]) impacts on modulation of neuronal signalling by inflammation. Conversely, inflammation of the lower urinary tract may impact on circulating estrogen levels or local estrogen production. Moreover, estrogens have a complex role in modulating inflammation [54], so the nature of cyclophosphamide-induced cystitis may not be the same in ovariectomised animals.

## Conclusions

This study has revealed novel patterns of activation of p38 and ERK MAP kinases in lumbosacral dorsal root ganglia following acute exposure *in vitro *or chronic deprivation of estrogens *in vivo*. The diversity of estrogen actions in these ganglia that have a major role in pelvic visceral pain raises the possibility of developing new ways to modulate their function in hyperactivity or pain states.

## Abbreviations

CGRP: calcitonin gene-related peptide; CREB: cyclic AMP response element binding protein; CYP: cyclophosphamide; DPN: diarylpropionitrile; DRG: dorsal root ganglion/ganglia; E2: 17β-estradiol, ER: estrogen receptor; ERK: extracellular signal-related kinase; MAP kinase: mitogen-activated protein kinase; NGF: nerve growth factor; OVX: ovariectomy; PBS: phosphate-buffered saline; PPT: propyl pyrazole triol; TRPV1: transient receptor potential vanilloid receptor 1

## Authors' contributions

YC carried out neuronal cultures, western blotting studies, cyclophosphamide treatment and ovariectomies. YC also participated in drafting the manuscript and figures. JK designed the study, performed the tissue dissections for *in vivo *studies, immunohistochemistry and microscopy. JK had primary responsibility for drafting the manuscript text and final figures. Both authors read and approved the final manuscript.
